# Vasopressin and its analogues in shock states: a review

**DOI:** 10.1186/s13613-020-0628-2

**Published:** 2020-01-22

**Authors:** Julien Demiselle, Nicolas Fage, Peter Radermacher, Pierre Asfar

**Affiliations:** 1 Service de Médecine Intensive et Réanimation, Médecine Hyperbare, Centre Hospitalier Universitaire, 4, Rue Larrey, 49933 Angers Cedex 9, France; 2 Institut für Anästhesiologische Pathophysiologie und Verfahrensentwicklung, Universitätsklinikum, Helmholtzstrasse 8-1, 89081 Ulm, Germany

**Keywords:** Vasopressin, Septic shock, Vasoplegia, Decatecholaminization

## Abstract

Activation of arginine–vasopressin is one of the hormonal responses to face vasodilation-related hypotension. Released from the post-pituitary gland, vasopressin induces vasoconstriction through the activation of V1a receptors located on vascular smooth muscle cells. Due to its non-selective receptor affinity arginine–vasopressin also activates V2 (located on renal tubular cells of collecting ducts) and V1b (located in the anterior pituitary and in the pancreas) receptors, thereby potentially promoting undesired side effects such as anti-diuresis, procoagulant properties due to release of the von Willebrand’s factor and platelet activation. Finally, it also cross-activates oxytocin receptors. During septic shock, vasopressin plasma levels were reported to be lower than expected, and a hypersensitivity to its vasopressor effect is reported in such situation. Terlipressin and selepressin are synthetic vasopressin analogues with a higher affinity for the V1 receptor, and, hence, potentially less side effects. In this narrative review, we present the current knowledge of the rationale, benefits and risks of vasopressin use in the setting of septic shock and vasoplegic shock following cardiac surgery. Clearly, vasopressin administration allows reducing norepinephrine requirements, but so far, no improvement of survival was reported and side effects are frequent, particularly ischaemic events. Finally, we will discuss the current indications for vasopressin and its agonists in the setting of septic shock, and the remaining unresolved questions.

## Background

Vasopressin (arginine–vasopressin, AVP, also called “antidiuretic hormone” ADH) is a natural hormone with potent vasoconstrictive effects. Its vasoconstrictive property was discovered in the nineteenth century by Oliver et al. after analysis of post-pituitary gland extracts [1]. While its vasoconstrictor effects are due to activation of V1a receptors, vasopressin also activates V2 and V1b as well as oxytocin receptors, thereby promoting anti-diuresis and exerting procoagulant activity. During the last three decades, its vasoconstrictor properties have prompted growing interest for its use in the management of vasodilatory shock.

In this review, we aim at describing the (patho)-physiological rationale for the use of AVP in distributive shock. Thereafter, we will report main results from animal and human studies, and describe benefits and risks associated with administration of vasopressin or its analogues. Finally, we will focus on vasopressin and its agonists use in the setting of septic shock and vasoplegic shock after cardiac surgery.

### Physiology of vasopressin and its receptors

Vasopressin is a nine-amino-acid peptide, synthesized in the hypothalamus as a “pre-pro-hormone”. This precursor circulates along axons of magnocellular neurons, through the pituitary stalk, to the post-pituitary gland. Vasopressin is stored in the post-pituitary gland, mostly in the intracellular compartment. After stimulation, only 10–20% of the amount of vasopressin can be immediately released into blood circulation.

Physiologically, plasma osmolality as well as blood volume and pressure are the main regulators of vasopressin secretion. Clearly, the former is the most important one: increased plasma osmolality sensed by hypothalamic osmoreceptors, leads to a pronounced increase in the plasma vasopressin level. A 2% decrease in whole body water leads to a doubling in vasopressin level [2].

The latter, via hypovolemia and hypotension, induces stimulation of atrial volume receptors and carotid baroreceptors, which results in vasopressin secretion. Secretion of vasopressin is more sensitive to small osmolarity variations as compared to hypotension-related vasopressin release that requires large pressure and volume variations [3].

Vasopressin has a short plasma half-life of 5–15 min, its clearance mainly depending on renal and liver vasopressinases [4].

In the periphery, vasopressin binds to 3 receptor subtypes, all belonging to the family of membrane-bound G protein-coupled receptors [5]:

- V1a receptors are located on vascular smooth muscle cells, their activation causes vascular smooth cell contraction.
- V2 receptors are located on basolateral surface of renal tubular cells, mainly on collecting ducts. Vasopressin binding induces aquaporin 2 recruitment, which allows an increase in permeability of epithelial membrane to water, leading to water re-absorption.
- V1b receptors are located in the anterior pituitary and in the pancreas. Vasopressin induces corticotropic axis stimulation (increase in cortisol) and insulin secretion.

**Fig. 1:**
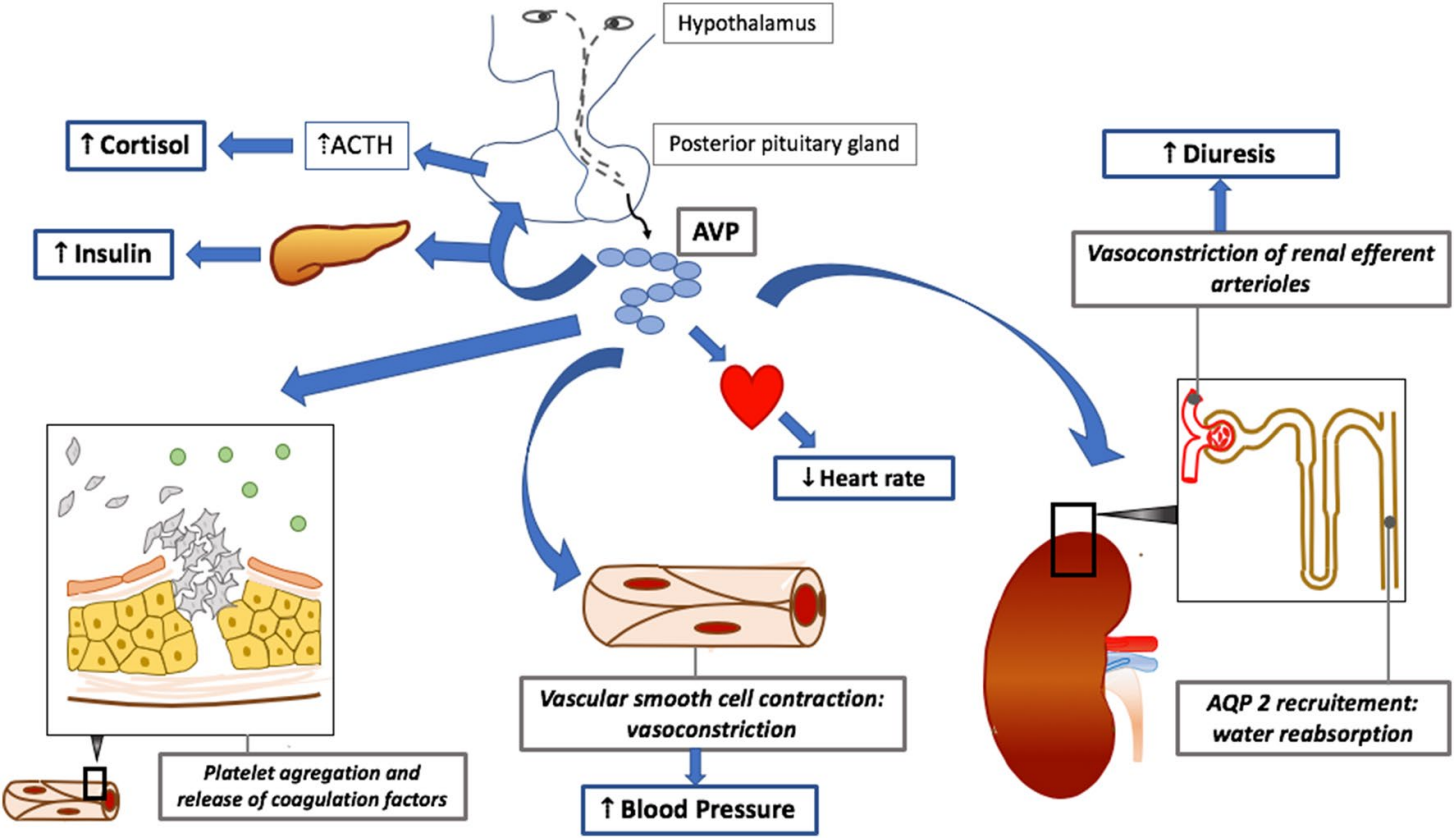
Physiological effects of vasopressin. *AVP* Arginine VasoPressin, *AQP2* Aquaporin 2. Vasopressin is synthesized in the hypothalamus and circulates along axons of magnocellular neurons to the post-pituitary gland. After stimulation, vasopressin is released into blood circulation, to 3 receptor subtypes. Binding on V1a receptors induces vascular smooth cell contraction in the periphery and on renal efferent arteriole and platelet aggregation. Vasopressin binding on renal V2 receptors causes aquaporin 2 recruitment, leading to water re-absorption and on extra-renal V2 receptors induces the release of coagulation factors. Binding on V1b receptors induces corticotropic axis stimulation and insulin secretion. During septic shock, vasopressin plasma level is low. Administration of vasopressin or its analogues induces a strong vasoconstriction, leading to an increase in blood pressure, and higher glomerular filtration rate

Of note, other functions have been described. Vasopressin has procoagulant property: activation of V1a receptors leads to platelet aggregation, and extra-renal V2 receptors activation induces the release of coagulation factors [6, 7]. In addition, oxytocin and vasopressin can cross-activate their respective receptors [8, 9]. Figure 1 summarizes the physiologic effects of vasopressin.

The ratio of V1/V2 effects reflects vascular selectivity. This ratio is close to 1 for vasopressin, in other words natural AVP has no receptor selectivity. Synthetic analogues, which have been tested in pre-clinical shock models and/or patients with vasodilatory shock of various causes, have higher vascular specificity. For example, terlipressin (Phe(2)-Orn(8)-vasotocin) has a V1/V2 ratio around 2.2 [10].

### Physiopathological mechanisms involved in septic shock

Septic shock is characterized by micro- and macrocirculatory disturbances with a decrease in peripheral vascular resistance, maldistribution of cardiac output to organs, and impairment of oxygen extraction. Several mechanisms contribute to sepsis-related vasodilatation, and vasodilatation occurs despite high endogenous catecholamines levels and activation of the renin–angiotensin–aldosterone system [11, 12].

The impact of vasopressin on the vasomotor tone is marginal in healthy subjects. During shock, blood vasopressin concentration is initially high. However, for similar levels of hypotension Landry et al. showed in 19 patients with septic shock that vasopressin plasma level was inappropriately low, as compared to 12 patients with cardiogenic shock [13]. Data regarding the natural course of vasopressin level was reported in a prospective cohort of 182 patients [14]: the more serious the infection (sepsis versus severe sepsis versus septic shock), the lower the vasopressin level was. In septic shock, after an initial and transient increase, plasma levels of vasopressin decreased until day 4 [15], which coincided with a decreased post-pituitary storage [16].

In addition to the deficiency of circulating vasopressin, Landry et al. also highlighted the hypersensitivity of patients with septic shock to exogenous vasopressin administration: while arterial hypotension was corrected [13, 17] in patients with septic shock, similar infusion rates had no effect on blood pressure in healthy subjects. All these observations together with the fact that exogenous vasopressin offers an alternative mechanism of action independent of adrenergic receptor activation prompted the interest for this hormone in the management of patients with septic shock [12].

### Haemodynamic effects of V1a agonist administration in septic shock

#### V1a agonists and cardiovascular system

In the experimental model, vasopressin administration resulted in various effects depending on the shock model and on vasopressin dose. When vasopressin was administered in hypodynamic endotoxin shock model, a decrease in cardiac output and myocardial ischaemia was observed [18–20]. In a hyperdynamic model of shock, the impact of vasopressin was different according to its infusion rate. High dose of vasopressin (greater than 0.15 UI/min) resulted in a decrease in cardiac output, oxygen consumption and in regional organ blood flow [21, 22]. When lower doses of vasopressin were tested (less than 0.1 U/min), mean arterial pressure was increased, without detrimental effect on cardiac output [23]. In another animal model (porcine faecal peritonitis) with infusion of low dose of vasopressin, heart rate and cardiac output were decreased without increase in troponin level nor change in myocardial relaxation, suggesting that vasopressin was safe regarding the theoretical risk of coronary vasoconstriction [24].

Exploratory clinical studies highlighted that vasopressin or terlipressin administration in septic shock patients allowed an increase in blood pressure and a decrease in norepinephrine requirement. The effect of vasopressin on cardiac output is more controversial. On one hand, non-randomized clinical studies reported a decrease in cardiac output [25, 26], on the other hand, a randomized study with small sample size did not confirm this finding. Finally, cardiac output, with similar target of blood pressure, was similar [27] or higher [28] in patients receiving vasopressin in addition to norepinephrine. Of note, vasopressin administration was safe regarding coronary circulation: its administration was not associated with differences between troponin serum levels, electrocardiogram patterns in a secondary analysis of a randomized controlled trial comparing norepinephrine and vasopressin in septic shock patients [29].

#### V1a agonists and splanchnic circulation

Because of its strong vasoconstrictor effect, concerns have been raised about vasopressin use and its impact on splanchnic circulation with a fear of splanchnic ischaemia [30] and liver dysfunction [31]. These arguments were turned down with experimental studies. It was demonstrated that with adequate fluid resuscitation, mesenteric flow and ileal microcirculation were preserved in a rat model of resuscitated endotoxin shock model with low doses of terlipressin [23]. These findings were in accordance with results of prospective randomized experimental study in a model of hyperdynamic porcine endotoxemia, where terlipressin administration was not associated with detrimental effect on hepatosplanchnic perfusion, oxygen exchange and metabolism [32]. Conversely, in an endotoxic pig model with high doses of vasopressin administration (0.28 UI/min), a decrease in splanchnic blood flow with an increased lactate release was observed [19]. Interestingly regarding safety concerns, clinical trials [28, 33] did not report side effects on splanchnic circulation with vasopressin administration.

#### V1a agonists and renal circulation

Distribution of V1a receptors in renal circulation is heterogeneous. The vasoconstrictor effect of vasopressin at low dose acts predominantly on renal efferent arterioles and has negligible effect on the afferent arterioles. This mechanism is related to a local phenomenon of nitrogen monoxide release [34, 35].

The efferent vasoconstriction induces a theoretical increase in glomerular renal perfusion pressure that results in higher glomerular filtration. Indeed, the first clinical studies confirmed this finding with increased diuresis and creatinine clearance in patients treated with vasopressin [25, 27].

#### V1a agonists and skin circulation

Vasopressin induces vasoconstriction of cutaneous vessels, with a dose–effect relationship. In a retrospective study, Dünser et al. reported ischaemic lesions of skin in almost one-third of patients (19/63) exposed to vasopressin [36]. Risk factors associated with the occurrence of ischaemic cutaneous lesions were overweight, high dose of norepinephrine, transfusion of platelets and fresh frozen plasma, history of peripheral arterial occlusive disease and the occurrence of septic shock. Only these last two items remained associated with the occurrence of cutaneous complications after multivariate analysis.

### Current knowledge of vasopressin and analogues use in the management of septic shock

#### Vasopressin

Administration of vasopressin with or without norepinephrine during septic shock has been studied in large-scale studies, which assessed whether vasopressin administration could result in an improvement in survival and renal function.

Russel et al. in the VASST study (VAsopressin in Septic Shock Trial) [37], compared the effect of vasopressin administration (*n* = 396) versus norepinephrine administration (*n* = 382) in patients with septic shock, in a randomized, double-blinded placebo-controlled study. The primary endpoint was mortality at day 28. An a priori stratification with respect to severity was integrated into the study design: severity was defined according to the norepinephrine infusion rates required to maintain arterial pressure, i.e. < vs ≥ 15 µg/min. The vasopressin infusion rate was 0.01–0.03 U/min. No differences were observed between groups in survival at day 28 and 90. Furthermore, there was no difference in organ failure occurrence between groups. Of note, safety profile was reassuring with no significant difference between groups in side effects occurrence. In contrast to the authors’ initial hypothesis that the more severe patients would benefit from the vasopressin treatment, outcome did not differ in the stratum of patients with norepinephrine doses ≥ 15 µg/min; in patients with norepinephrine infusion rates < 15 µg/min, vasopressin administration was associated with significantly improved survival at day 28 (26.5% vs 35.7%, *p* = 0.05) and 90 (35.8% vs 46.1%, *p* = 0.04). It remains an open question, whether this effect was due to vasopressin per se and/or the fact that in the less severe patients, a higher proportion could be completely weaned from norepinephrine support [38]. A post hoc analysis of VASST study according to the Sepsis-3 definition confirmed this finding [39]: while in patients, who fulfilled the sepsis-3 criteria of septic shock, i.e. arterial hypotension requiring introduction of catecholamine to maintain mean blood pressure > 65 mmHg despite adequate fluid resuscitation and hyperlactatemia > 2 mmol/L, there was no difference in mortality, survival was increased in patients with hypotension requiring vasopressors alone without hyperlactatemia.

A further post hoc analysis of the VASST trial suggested an association between the use of vasopressin and hydrocortisone: vasopressin reduced mortality in patients, who—at the discretion of the attending physician—were treated with hydrocortisone. Conversely, in patients who did not received hydrocortisone, vasopressin administration was associated with higher mortality [40].

Another post hoc analysis of the VASST trial tried to assess the impact of vasopressin on kidney (dys)function: Gordon et al. analysed the 464 patients with acute kidney injury of VASST study cohort [41], categorizing them according to RIFLE classification [42]. Patients at “Risk” stage and treated with vasopressin had significantly less worsening of renal function (progression to “injury/failure” stage) than patients treated with norepinephrine.

The potential impact of hydrocortisone treatment together with a putative beneficial effect of vasopressin on AKI prompted the design of the VANISH study [43], which compared the effects of either vasopressin or norepinephrine as the vasopressor of first choice together with hydrocortisone or vehicle using a two-by-two factorial design. No interaction was identified between vasopressin and hydrocortisone [43]. Furthermore, neither mortality nor AKI were significantly influenced; nevertheless, the authors concluded that “*the confidence interval included a potential clinically important benefit for vasopressin*”, which should be tested further in larger trials.

Clearly, both the VANISH and VASST trials were “negative” in the sense that no superiority of vasopressin could be demonstrated. Nevertheless, these studies showed that vasopressin is not inferior to norepinephrine either, and, that its administration was not associated with higher rates of adverse effects, especially ischaemic events. Moreover, these studies demonstrated that vasopressin allows at least a reduction of norepinephrine infusion rates and fastened weaning from norepinephrine. In the context of “decatecholaminization”, vasopressin use limits norepinephrine infusion rates and potentially its related side effects [44]. Interestingly, in a retrospective study, patients with septic shock who received fixed-dose of vasopressin in association with norepinephrine had an improve prognosis when vasopressin infusion resulted in an increase in mean arterial pressure [45].

#### Vasopressin analogues: terlipressin and selepressin

Synthetic analogues of vasopressin with higher vascular selectivity (V1/V2 ratio > 1) were also studied in the setting of septic shock.

In a blinded, randomized study recruiting 32 patients with septic shock, terlipressin administration in combination with norepinephrine was superior to norepinephrine alone (complication and survival at day 7), but results have to be tempered due to the small sample size [46].

In 2018, Liu et al. reported results of a randomized multicentric double-blind study, aimed at comparing terlipressin and norepinephrine as first-line vasopressor agent in septic shock [47]. In this study, 526 patients were analysed: 260 received up to 4 mg terlipressin per day, and 266 received norepinephrine. There was no difference in mortality at day 28 between groups (primary endpoint). Likewise, improvement and variation of SOFA score until day 7 were comparable. Terlipressin administration was associated with an increase in adverse effects (30% vs 12%, *p* < 0.01). The most common was digital ischaemia (12.6% in terlipressin group versus 0.35% in norepinephrine group, *p* < 0.001). 76% of adverse effects occured during the first 24 h.

A more recent synthetic V1a agonist, selepressin, was reported, in a sheep model of faecal peritonitis, to be superior to norepinephrine and vasopressin. If, in established septic shock, selepressin maintained mean arterial pressure to a similar extent as did vasopressin and norepinephrine, selepressin administration resulted in reduced lung edema [48]. These results were in accordance with previous study, highlighting the reduced vascular leakage with selepressin administration [49].

Recently, the SEPSISACT study [50] compared with a randomized placebo-controlled trial the addition of selepressin to norepinephrine. This study was divided in two parts: the first was aimed at assessing the best-performing regimen of selepressin (between three dosing regimens of selepressin), and, in the second part, the authors compared this selepressin regimen to placebo. Among the 817 patients who completed the trial, no difference between the two groups on the primary composite endpoint was observed (ventilator and/or vasopressor free days within 30 days). Likewise, no difference in secondary endpoints was observed (mortality, need for renal replacement therapy and length of stay in the intensive care unit). Selepressin administration was not associated with higher rate of side effects.

A meta-analysis of 20 study including patients with septic shock suggested a potential improvement of survival with the use of vasopressin (9 studies) and its analogues (11 studies), when compared with adrenergic vasopressors (mostly norepinephrine). This meta-analysis underlined the additional risk of digital ischaemia [51]. In a recent meta-analysis of individual patients data from randomized studies (1453 septic shock patients) [52], no difference in mortality was found between vasopressin and norepinephrine. Furthermore, vasopressin use was not associated with more undesirable effect, but safety profiles were different: vasopressin led to more digital ischaemia, but fewer arrhythmias. This decrease in the incidence of arrhythmias was also reported in a meta-analysis dedicated to this specific question [53]. In this analysis were included randomized controlled trials aimed at comparing the association of vasopressin with norepinephrine to catecholamines alone for patients with distributive shock.

### Current knowledge of vasopressin and analogues use in the management of vasoplegic shock after cardiac surgery

Furthermore, vasopressin was evaluated in the setting of vasoplegic shock after cardiac surgery. In 2003, among 48 patients with vasoplegic shock following cardiac surgery, a combination of vasopressin and norepinephrine versus norepinephrine alone showed an improvement of haemodynamic parameters, and fewer new-onset tachyarrhythmias when vasopressin was associated [28].

In VANCS trial [54], a randomized controlled monocentric study, patients in post-operative of cardiac surgery with refractory hypotension after fluid resuscitation, without cardiac output impairment (> 2.2 L/min/m^2^), received vasopressin (0.01 at 0.06 U/min) or norepinephrine (10 at 60 µg/min). The primary composite endpoint (death or major complication in the 30 post-operative days) occurred more frequently in norepinephrine group than in the vasopressin group. Interestingly, patients treated with vasopressin presented less new-onset tachyarrhythmias. There were no harm effects (digital, myocardial, mesenteric ischaemia nor hyponatremia) in the vasopressin group.

## Conclusion: what is the current place for vasopressin and its analogues in the management of vasoplegic shock states?

Recommendations of Surviving Sepsis Campaign suggest administration of vasopressin in second-line after norepinephrine, to maintain mean blood pressure goal or to decrease norepinephrine dosage [55].

Use of vasopressin or analogue in first line is marginal [56], and its use has never been associated with improved prognosis of patient in septic shock.

After this review of current knowledge, we might suggest that vasopressin and analogues could be considered:

- Early, in patient with sepsis with low blood pressure, without hyperlactatemia, alone or in association with norepinephrine.
- In association with norepinephrine, to decrease norepinephrine infusion rate in order to limit the undesirable effects, according to the concept of “decatecholaminization” [57].

## Data Availability

Not applicable.
